# Authors' reply

**DOI:** 10.4103/0972-9941.38914

**Published:** 2007

**Authors:** Arshad M Malik, A A Laghari, K A Talpur, A Memon, Q Mallah, J M Memon

**Affiliations:** Department of Surgery, Liaquat University of Medical and Health Sciences, Jamshoro, Pakistan. E-mail: arshadhamzapk@yahoo.com

Dear sir,

Follwing are answers for comments[1] on out published article in Journal of Minimal Access Surgery.[2]

## How was the gall bladder extracted? Was an extraction bag used?

All the infecteg gall bladders were retrieved by using an endo bag.

## The role of analysis by SPSS version 10 has not been further elucidated anywhere in the text. What variable was being assessed?

The demographic details and various other variables like hospital stay,duration of surgery etc are all alnalysed on SPSS.

## In the exclusion criteria did the authors exclude patients with bleeding disorders?

Fortunately none of the study subject had derainged coagulation profile otherwise it must have been corrected before surgery.

## The USG findings discussed are non specific and usually it is quite difficult to predict empyema gall bladder with these findings. We have found out that a thick walled gall bladder >4mm with a stuck stone at the neck is more of a predictor of empyema than other findings mentioned. Even the clinical features are sometimes misleading.

Very true as there are no fixed ultrasonographic criteria to label a case as empyma pre-operatively but the investigations are complementry to clinical findings.we found empyma on laparoscopy in majority of patients.

## In table 3A the number of CBD injuries shown is 3 while as in the discussion repair of only two cases is documented. Which one is correct?

Two cases had bile duct injury.Five cases of gallbladder perforation occurred instead of four.

## After conversion what were the results? Was cholecystectomy completed in all patients?

All converted patients were completed successfully

## What is the role of intra operative cholangiography in such cases?

The role of intra-operative cholangiography in such difficult situation is long debated and there is a consensus about its usefulness but unfortunately we do not have this facility in our institute.

## What is the role of laparoscopic cholecystostomy in empyema gallbladder? Would it have decreased the overall conversion and the complication rates?

Earlier studies documented an overall increased conversion rate but that was ascribed to learning curve and subsequent studies have proved that it is no longer a contra-indication to laparoscopic approach. Same is true for the complications occurring during laparoscopic cholecystectomy in such acute cases.

## Did the very high morbidity documented justify the early intervention in pre surgery documented cases of empyema?

Yes but the experience of the operating surgeon plays a vital role in this context.It is very clear that such cases are not to be done by the beginners as there can be life threatening complications.
